# Diterpenoids inhibit ox-LDL-induced foam cell formation in RAW264.7 cells by promoting ABCA1 mediated cholesterol efflux

**DOI:** 10.3389/fphar.2023.1066758

**Published:** 2023-01-12

**Authors:** Cheng Zhang, Xuewen Wu, Pengmin Shi, Hongyu Ma, Fei Fang, Qianlang Feng, Shuang Zhao, Ruipu Zhang, Jinyuan Huang, Xinting Xu, Weilie Xiao, Guang Cao, Xu Ji

**Affiliations:** ^1^ Key Laboratory of Medicinal Chemistry for Natural Resource, Ministry of Education, Yunnan Provincial Center for Research and Development of Natural Products, Yunnan Characteristic Plant Extraction Laboratory, Ministry of Education, School of Pharmacy, Yunnan University, Kunming, China; ^2^ Department of Pulmonary and Critical Care Medicine, Xi’an International Medical Center Hospital, Xi’an, China

**Keywords:** diterpenoids, atherosclerosis, foam cell, cholesterol efflux, reverse cholesterol transport

## Abstract

**Introduction:** Atherosclerosis is the main cause of many cardiovascular diseases and contributes to morbidity and mortality worldwide. The formation of macrophage-derived foam cells plays a critical role in the early stage of atherosclerosis pathogenesis. Diterpenoids found in the flowers of *Callicarpa rubella* Lindl., a traditional Chinese medicine, have been reported to have anti-inflammatory activity. However, little is known about the effects of these diterpenoids on macrophage foam cell formation.

**Methods:** A macrophage-derived foam cell formation model was established by treating RAW264.7 cells with oxidized low-density lipoprotein (ox-LDL) for 24 h. Oil red O staining were used to detect the intracellular lipids. The cholesterol efflux capacity was assayed by labeling cells with 22-NBD-cholesterol. Western blots and real-time PCRs were performed to quantify protein and mRNA expressions.

**Results:** Two diterpenoid molecules, 14α-hydroxyisopimaric acid (C069002) and isopimaric acid (C069004), extracted from the flowers of *Callicarpa rubella* Lindl., significantly attenuated ox-LDL-induced foam cell formation in RAW264.7 macrophages. Further investigation showed that these two diterpenoids could promote cholesterol efflux from RAW264.7 macrophages to apolipoprotein A-I or high-density lipoproteins, which was associated with upregulated expression of ATP-binding cassette A1/G1 (ABCA1/G1), liver X receptor-α (LXRα), and peroxisome proliferator-activated receptor-γ (PPARγ). Unexpectedly, the diterpenoids C069002 and C069004 failed to enhance the mRNA transcription of the ABCG1 gene in macrophage-derived foam cells induced by ox-LDL. To evaluate the effects of diterpenoids on macrophage foam cell formation and determine the underlying mechanism, two drugs (lovastatin and rosiglitazone) were used as positive controls. Although both drugs could reduce macrophage foam cell formation and promote cholesterol efflux, they each had distinctive abilities to modulate the expression of cholesterol efflux-related genes. In contrast to lovastatin, rosiglitazone showed a similar influence on the expression of cholesterol efflux-related genes (including *ABCA1*, *LXRα*, and *PPARγ*) as the diterpenoids regardless of the presence or absence of ox-LDL, implying a similar mechanism by which they may exert atheroprotective effects.

**Conclusion:** Our research indicates that diterpenoids effectively inhibit ox-LDL-induced macrophage foam cell formation by promoting cholesterol efflux from macrophages via the PPARγ-LXRα-ABCA1 pathway. Further investigation of diterpenoids as potential drugs for the treatment of atherosclerosis is warranted.

## 1 Introduction

Atherosclerosis or coronary artery disease is the most common form of cardiovascular disease (CVD) triggered by lipid accumulation and inflammation in the arteries, which may eventually lead to clinical complications, myocardial infarction (MI) and stroke. As a chronic inflammatory disease, clinically significant atherosclerosis occurs primarily in older individuals and is the leading cause of mortality and morbidity worldwide. Atherosclerosis is initiated largely by the accumulation of certain plasma lipoproteins, including low-density lipoproteins and remnants of triglyceride-rich lipoproteins, in the intimal region of the vessel. The lipoproteins then aggregate and become oxidized and otherwise modified, resulting in the activation of the overlying endothelial cells to express adhesion and chemotactic molecules for monocytes. Blood monocytes then enter the intima, differentiate into macrophages, and take up modified lipoproteins through scavenger receptors or phagocytosis of aggregated lipoproteins to give rise to cholesterol-engorged macrophages or “foam cells” (Bjorkegren and Lusis, 2022). Foam cells are a hallmark of atherosclerosis. They play an important role at all stages of atherosclerotic lesion development, from the initial lesions to the stability of advanced plaques (Koelwyn et al., 2018). In advanced atherosclerotic lesions, foam cells frequently undergo apoptosis or necrosis to give rise to a growing “necrotic core” consisting of cholesterol esters, cholesterol crystals, and cell debris, which increases the likelihood of lesion rupture (Engelen et al., 2022). If this happens in the coronary arteries, it can generate a local clot that may completely obstruct the blood flow to cause an MI. Alternatively, the clot can escape the heart and travel to the brain, where it may cause a stroke (Fan and Watanabe, 2022). Therefore, inhibiting macrophage-derived foam cell formation may be a promising strategy to prevent and attenuate atherosclerosis.

Reverse cholesterol transport (RCT) is a process by which excessive cholesterol excreted from cells is transported to the liver through the circulation for metabolism and is finally excreted through bile and feces (Rader et al., 2009). It plays vital roles in maintaining intracellular lipid homeostasis, restraining foam cell formation, and protecting against atherosclerotic CVD (Ouimet et al., 2019). The initial and key step of RCT is cholesterol efflux from nonhepatic peripheral tissues (e.g., macrophages and vascular smooth muscle cells) to extracellular lipid acceptors, resulting in high-density lipoprotein (HDL) formation (Pownall et al., 2021). During this process, several lipid transporters have been shown to promote cholesterol efflux *in vitro* and *vivo*, and the most critical transporters are ATP-binding cassette (ABC) transporter A family member 1 (ABCA1) and ABC transporter G family member 1 (ABCG1). ABCA1 promotes cholesterol efflux to the free apolipoprotein A-I (ApoA-I) of nascent HDL, while ABCG1 mediates cholesterol efflux from macrophages to mature HDL particles (Li et al., 2021; Dergunov and Baserova, 2022). Previous studies have shown that ABCA1 gene mutation or suppression of ABCG1 expression could decrease cholesterol efflux from lipid-laden macrophages to ApoA-I or HDL, respectively (Rust et al., 1999; Klucken et al., 2000). Moreover, combined deficiency of ABCA1 and ABCG1 promotes foam cell accumulation and accelerates atherosclerosis in mice (Yvan-Charvet et al., 2007). Thus, enhancement of ABCA1 and ABCG1 expression can greatly alleviate macrophage lipid deposition and atherogenesis. Macrophage expression of ABCA1 and ABCG1 is regulated by liver X receptor α (LXRα), which acts as a heterodimer with its partner, the retinoid X receptor (RXR) (Rader et al., 2009). LXRα, a ligand-activated nuclear transcription factor, is a member of the nuclear receptor superfamily and is involved in regulating cholesterol metabolism and lipid biosynthesis. Activation of LXRα initiates the transcription of multiple genes, participates in the RCT pathway, maintains lipid metabolism balance, and promotes cholesterol outflow. The main downstream target genes of LXRα are *ABCA1*, *ABCG1*, *SREBP-1c*, *apoE*, and phospholipid transporters (Zhang et al., 2021). Binding of ligand to LXRα results in the upregulation of ABCA1 and ABCG1 transcription in macrophages, enhancement of cholesterol efflux, and inhibition of atherosclerosis progression (Zeng et al., 2018; Joseph et al., 2002). In addition, many studies have claimed that another ligand-activated nuclear receptor, namely, peroxisome proliferator-activated receptor γ (PPARγ), participates in the regulation of ABCA1/G1 expression and lipid metabolism in macrophages (Liu et al., 2021; Wu et al., 2021; Wang et al., 2022). PPARγ agonists or activators can promote macrophage cholesterol efflux and protect against atherosclerosis by augmenting the expression of LXRα and ABCA1/G1 (Chawla et al., 2001; Ren et al., 2019; Du et al., 2021). Accordingly, activation of the macrophage PPARγ-LXRα-ABCA1/G1 pathway may be a promising therapeutic strategy against exacerbation of atheroma lesions.

As traditional Chinese folk medicines, the genus *Callicarpa* (family Lamiaceae) is widely distributed in southern China and has been regularly used to treat inflammation (Wang et al., 2019), abscesses, and bacterial infection (Pineau et al., 2019). One of the major structures of the chemical constituents in plants of the genus *Callicarpa* is diterpenoids. Some diterpenoids extracted from the flowers of *Callicarpa rubella* Lindl. have been shown to reduce the lipopolysaccharide (LPS)-induced inflammatory response in macrophages (Li et al., 2020; Wu et al., 2020). However, the antiatherogenic effects of diterpenoids have rarely been mentioned. Thus, in this study, we aimed to uncover the role of diterpenoids in macrophage foam cell formation in the early stage of atherosclerosis. Our investigation showed that diterpenoids attenuated ox-LDL-induced foam cell formation in RAW264.7 macrophages by promoting cholesterol efflux *via* the PPARγ-LXRα-ABCA1 signal transduction pathway.

## 2 Materials and methods

### 2.1 Reagents

Lovastatin (Cat No. SL8280) and rosiglitazone (Cat No. R8470) were obtained from Solarbio Life Sciences (Beijing, China). Ox-LDL (Cat No. YB-002) was purchased from Yiyuan Biotechnology (Guangzhou, China). Oil red O powder (Cat No. O0625) was purchased from Sigma (St. Louis, MO, United States). TRIzol reagent (Cat No. 15596026) (Invitrogen) was purchased from Thermo Fisher Scientific (Waltham, MA, United States). Total cholesterol (TC) (Cat No. E1015) and triglyceride (TG) (Cat No. E1013) assay kits were obtained from Applygen (Beijing, China). M-MLV reverse transcriptase (Cat No. M1701) and Eastep qPCR master mix (Cat No. LS 2062) were purchased from Promega (Madison, WI, United States). 22-NBD-cholesterol (Cat No. 625332) was purchased from J&K Scientific (Beijing, China). Thiazolyl blue tetrazolium bromide (MTT) (Cat No. M8180) was purchased from Solarbio Life Sciences (Beijing, China). Cell Counting Kit-8 (CCK-8) (Cat No. PF00004) and the enhanced chemiluminescence (ECL) reaction kit (Cat No. PK10001) was purchased from Proteintech (Rosemont, IL, United States). Pierce bicinchoninic acid (BCA) protein assay kit (Cat No. 23227) was purchased from Thermo Fisher Scientific (Waltham, MA, United States). Rabbit polyclonal antibodies against ABCG1 (Cat No. 13578-1-AP), PPAR gamma (Cat No. 16643-1-AP), and NR1H3 (LXR alpha) (Cat No. 14351-1-AP) were purchased from Proteintech (Rosemont, IL, United States). The rabbit monoclonal antibody against ABCA1 (Cat No. 96292S) was obtained from Cell Signaling Technology. The mouse monoclonal antibody against β-actin (Cat No. sc-47778), horseradish peroxidase (HRP)-conjugated anti-rabbit IgG secondary antibody (Cat No. sc-2357), and HRP-conjugated anti-mouse IgG secondary antibody (Cat No. sc-516102) were purchased from Santa Cruz Biotechnology (Santa Cruz, CA, United States). The RAW264.7 cell line (Cat No. TIB-71) (ATCC, Manassas, VA, United States) was obtained from Prof. Xiao’s laboratory. ApoA-I (Cat No. 10686-H02H) was obtained from Sino Biological (Beijing, China). HDL (Cat No. H7940) was purchased from Solarbio Life Sciences (Beijing, China).

### 2.2 Plant material

The flowers of *Callicarpa rubella* Lindl. [Lamiaceae] were collected from Xingyi, Guizhou Province, P. R. China, during April 2016 and identified by Prof. Xiwen Li. A voucher specimen (YNUXWL 20160421) was stored at the Key Laboratory of Medicinal Chemistry for Natural Resource, Ministry of Education, Yunnan University.

### 2.3 Extraction and isolation of the diterpenoids from *C. rubella*

The diterpenoid molecules (C069002 and C069004) were obtained from the flowers of *C. rubella* by various chromatographic methods and the details were described below: The air-dried powder of the flowers of *C. rubella* (7.5 kg) was extracted three times (20 L, each) with 80% methanol at room temperature to give a crude extract, which was suspended in H_2_O and extracted with ethyl acetate (4 × 3 L) to collect ethyl acetate (EtOAc) solution (520 g). The EtOAc extract was subjected to silica gel column chromatography and eluted using a gradient petroleum ether-ethyl acetate (PE–EtOAc) (10:1 to 0:1, V/V) system, followed by elution with methanol to yield fractions 1–5. Fraction 4 (110 g) was subjected to MCI gel column and eluted with methanol–H_2_O system (6:4 to 10:0) to obtain five fractions (Fr. 4.1–Fr. 4.5). Fr. 4.2 (10.0 g) was separated by silica gel column chromatography, eluted with PE–EtOAc (10:1 to 0:1, V/V) to get five fractions (Fr. 4.2b1 to Fr. 4.2b5). Fr. 4.2b2 was purified by petroleum ether–acetone (3:1) on silica gel to afford compound **C069002** (obtained as colorless crystals). While, Fraction 3 (50 g) was applied to silica gel column chromatography (PE–EtOAc, 20:1 to 1:1, V/V) to obtain five fractions (Fr. 3.1–Fr. 3.5) together with the crystals of **C069004**.

### 2.4 Identification of the diterpenoids from *C. rubella*

The diterpenoid molecules (C069002 and C069004) were obtained as colorless crystals and their molecular formulas were determined to be C_20_H_30_O_3_ and C_20_H_30_O_2_ respectively based on high resolution electrospray ionization mass spectroscopy (HR-ESI-MS) data. Further interpretation of their nuclear magnetic resonance (NMR) data indicated that its structure resembled highly that of pimarane diterpenoids (Supplementary Figure S1). And their structures were confirmed by comparison their NMR signals with those published in the literature (Muto et al., 2008; Ryu et al., 2010).

### 2.5 Oil red O staining

Cellular lipid accumulation was evaluated by oil red O staining. In brief, RAW264.7 macrophages cultured in Dulbecco’s modified eagle medium (DMEM) containing 10% fetal bovine serum (FBS) and 1% antibiotic (100 U/mL penicillin and 100 μg/mL streptomycin) were seeded on 24-well plates at 1×10^7^ cells/mL or 5×10^5^ cells/mL. After 12 h, when the cells had grown to 75%–80% confluence, they were exposed to different concentrations of ox-LDL or ox-LDL (80 μg/mL) combined with the indicated drugs (lovastatin, rosiglitazone, C069002, and C069004) at various concentrations for 12, 24 or 48 h. Then, the cells were washed with phosphate buffered saline (PBS) and fixed with 10% formaldehyde, followed by dehydration with 60% isopropanol for 15 s. The lipids were stained using filtered fresh oil red O solution for 15 min at 37°C. Subsequently, the cells were viewed using an optical microscope (Olympus, Japan), and the lipid droplet content was measured using a spectrophotometer (SpectraMax M2, Molecular Devices) at 510 nm absorbance.

### 2.6 Cell viability and cell proliferation assays

The 3-(4,5-dimethylthiazol-2-yl)-2,5-diphenyltetrazolium bromide (MTT) assay was applied to assess cell viability as previously described (Kumar et al., 2018). RAW264.7 cells (1×10^5^ cells/well) were seeded in 96-well plates in culture medium. After 24 h of culture, the cells were treated with various concentrations of lovastatin, rosiglitazone, and diterpenoids (C069002 and C069004) for another 24 h. Subsequently, the cells were washed with culture medium and further incubated with 5 mg/mL MTT at 37°C for 4 h. Next, the culture medium was removed, and 100 μL of DMSO was added to the wells. Finally, the purple formazan dye aggregates in the cell were detected using a microplate reader (SpectraMax M2, Molecular Devices) at 490 nm absorbance. The effect of ox-LDL on the cell viability of RAW264.7 cells was determined by using the Cell Counting Kit-8 (CCK-8) according to the manufacturer’s instructions. To test the effect of ox-LDL on the proliferation of RAW264.7 cells, cells (1×10^5^ cells/well) were seeded in 12-well plates in culture medium. After 24 h of culture, the medium was removed, and 1 mL of new medium containing 80 μg/mL ox-LDL was added (marked as time 0 h) for subsequent continuous culture until 48 h. The cell amounts at 0, 12, 24, and 48 h were counted by using a hemocytometer.

### 2.7 TC and TG content assays

The levels of intracellular TC and TG in RAW264.7 cells were assayed using a TC assay kit and TG assay kit, respectively. RAW264.7 cells were seeded on 12-well plates (5 ×10^5^ cells/well) and exposed to ox-LDL (80 μg/mL) with or without 15 μmol/L or 30 μmol/L indicated compounds for 24 h. The cell lysate was incubated at 70°C for 10 min and then centrifuged at ×2,000 g for 5 min at 4°C. After that, the supernatant was transferred to measure the TC and TG contents by using a microplate reader (SpectraMax M2, Molecular Devices) at a wavelength of 550 nm. The standard curves of cholesterol and triglyceride were established by serial dilution of the cholesterol and triglyceride reference standards.

### 2.8 Cholesterol efflux assay

RAW264.7 cells were seeded on 12-well plates (1×10^6^ cells/well). After 12 h, when the cell confluence had reached 70%–80%, the medium was removed, and the cells were labeled with 22-NBD-cholesterol (final concentration of 5.0 μmol/L) in serum-free medium containing .2% (w/v) bovine serum albumin (BSA) (Solarbio Life Sciences) for 24 h in a 37°C 5% CO_2_ incubator. After 24 h of labeling, the cells were washed twice with PBS and treated with 15 μmol/L lovastatin, rosiglitazone, C069002, and C069004 for an additional 18 h. Thereafter, 10 μg/mL ApoA-I or 50 μg/mL HDL was added as the receptor protein to start the efflux experiment at 37°C for another 6 h. Finally, the amounts of cholesterol in the medium and cells were assayed using a microplate reader (excitation 485 nm, emission 535 nm). The percentage of 22-NBD-cholesterol (%) was calculated as (medium)/(medium + cell) × 100. Each efflux assay was performed in duplicate three times.

### 2.9 Western blotting

RAW264.7 macrophages were seeded on 6-cm Petri dishes at 6×10^5^ cells/mL. The cells were treated with 15 μmol/L lovastatin, rosiglitazone, C069002, and C069004 for 24 h. Thereafter, the cells were collected and resuspended in radioimmunoprecipitation assay (RIPA) lysis buffer supplemented with protease inhibitors. Then, the total protein was extracted from the cell lysate, and the protein concentration was determined by a Pierce BCA protein assay kit (Cat No. 23227) (Thermo Fisher Scientific, Waltham, MA, United States). Equal amounts of protein were loaded on 10% sodium dodecyl sulfate-polyacrylamide gels for electrophoresis and then transferred to .45-μm polyvinylidene fluoride membranes (Cat No. IPVH00010) (Millipore Corp., Bedford, MA, United States). The membranes were blocked with 5% (w/v) skimmed milk in tris-buffered saline containing .1% Tween-20 (TBST) for 2 h and then were incubated with the following primary antibodies diluted in blocking buffer overnight at 4°C: rabbit anti-PPARγ (1:2000), anti-LXRα (1:2000), anti-ABCA1 (1:500), anti-ABCG1 (1:500), and anti-β-actin (1:5000). Next, the membranes were washed three times with TBST buffer, followed by incubation with HRP-conjugated anti-mouse and anti-rabbit IgG secondary antibodies (1:5000) for 1 h at room temperature. Finally, after being extensively washed with TBST buffer, the protein bands were detected with an enhanced chemiluminescence (ECL) reaction kit (Cat No. PK10001) (Proteintech, Rosemont, IL, United States), and the signals were acquired by an enhanced ECL western blotting system (Tanon, Shanghai, China). The images were analyzed using Fiji software, which is available on the website (https://imagej.net/downloads) for free. All the proteins were normalized to the internal control (β-actin).

### 2.10 Quantitative real-time polymerase chain reaction (RT-PCR)

RAW264.7 macrophages were seeded on 6-cm Petri dishes at 6×10^5^ cells/mL. Then, the cells were treated with 15 μmol/L lovastatin, rosiglitazone, C069002 or C069004 in the presence or absence of 80 μg/mL ox-LDL for 24 h. Thereafter, the cells were harvested, and total RNA was extracted from the cells using TRIzol reagent (Cat No. 15596026) (Invitrogen, Waltham, MA, United States) according to the manufacturer’s instructions. Subsequently, first-strand cDNA was synthesized from total RNA using the M-MLV reverse transcriptase (Cat No. M1701) (Promega, Madison, WI, United States), and a real-time quantitative PCR assay with SYBR Green detection chemistry was performed on an ABI Q3 Real-Time PCR Detection System (ABI, United States). The sequences of the primers are listed in Table 1. Melting curves were recorded, and the specificity of the PCR products was checked by agarose gel analysis. The mRNA levels of all genes were normalized to the internal control (β-actin) levels, and the quantitative measurements were carried out by the ^ΔΔ^C_t_ method.

**TABLE 1:** Primers for real-time quantitative PCR.

Gene	Forward primer	Reverse primer
*ABCA1*	5′-CCC​TGC​TAC​ACT​GGT​CAT​TAT​C-3′	5′-CCC​ATA​CAG​CAA​GAG​CAG​AA-3′
*ABCG1*	5′-GTG​ACG​CTG​ACT​ATA​AGA​GA-3′	5′-AGG​TGA​TTC​GCA​GAT​GTG-3′
*LXRα*	5′-AGG​AGT​GTC​GAC​TTC​GCA​AA-3′	5′-CTC​TTC​TTG​CCG​CTT​CAG​TTT-3′
*PPARγ*	5′-GCA​TCT​CCA​CCT​TAT​TAT​TCT​G-3′	5′-CTT​CAA​TCG​GAT​GGT​TCT​TC-3′
*β-actin*	5′-ACC​ACA​CCT​TCT​ACA​ATG​AG-3′	5′-CGT​TGC​CAA​TAG​TGA​TGA​C-3′

### 2.11 Statistical analysis

Statistics were calculated with OriginPro version 8.0 software (OriginLab Corporation, Northampton, MA, United States). The data are presented as the mean ± SD or mean ± SEM. Differences between groups were determined by one-way ANOVA analysis by SPSS version 16.0 software (SPSS Inc., Chicago, IL, United States). All *p* values <.05 were considered statistically significant (^*^ *p* < .05, ^**^ *p* < .01; ^#^ *p* < .05, ^##^ *p* < .01; ^Δ^ *p*<.05, ^ΔΔ^ *p*<.01). The OriginPro version 8.0 software and SPSS version 16.0 software were obtained from the Library of Yunnan University.

## 3 Results

### 3.1 Diterpenoids alleviate ox-LDL-induced macrophage foam cell formation

**FIGURE 1:**
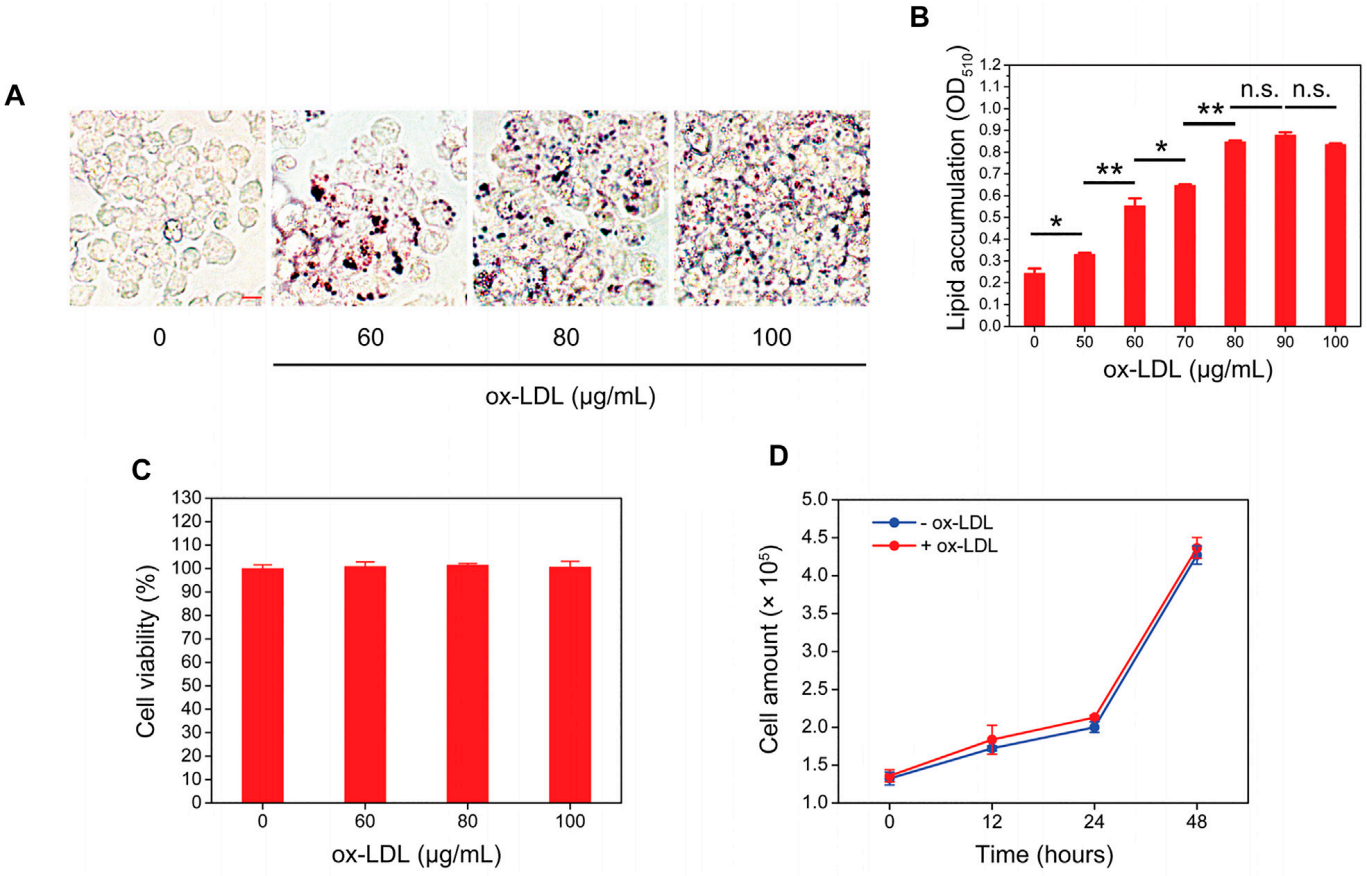
Ox-LDL induces the formation of foam cells by RAW264.7 macrophages. **(A)** RAW264.7 macrophages were incubated with ox-LDL at different concentrations for 24 h. After that, the intracellular lipid droplet content (red color) in RAW264.7 cells was determined by oil red O staining. Representative images of four individual ox-LDL concentrations (0, 60, 80, and 100 μg/mL) are shown (×400 magnification). Scale bar = 10 μm **(B)** Quantitative results of different ox-LDL treatments were obtained by measuring the eluted oil red O dye at 510 nm using a spectrophotometer. **(C)** RAW264.7 cells were incubated with different concentrations of ox-LDL for 24 h, and cell viability was measured by using a Cell Counting Kit-8 (CCK-8). **(D)** RAW264.7 cells were treated with 80 μg/mL ox-LDL for 48 h. At each indicated time point, the cell number was counted by using a hemocytometer. Values represent the mean ± SEM from three independent experiments. Significance is presented as *p < .05, **p < .01 between the indicated groups. n. s.: no significance.

A macrophage-derived foam cell formation model was initially established by treating RAW264.7 cells with different concentrations of ox-LDL for 24 h. Oil red O staining showed that ox-LDL increased the intracellular lipid content in a concentration-dependent manner when the concentration of ox-LDL lower than 80 μg/mL (Figures 1A, B). However, when the concentration of ox-LDL exceeded 80 μg/mL, the extent of intracellular lipid accumulation was no longer obviously aggravated (Figure 1B). In addition, we also evaluated the effects of ox-LDL on the viability and proliferation of RAW264.7 cells and found that ox-LDL did not affect cell viability or proliferation (Figures 1C, D).

**FIGURE 2:**
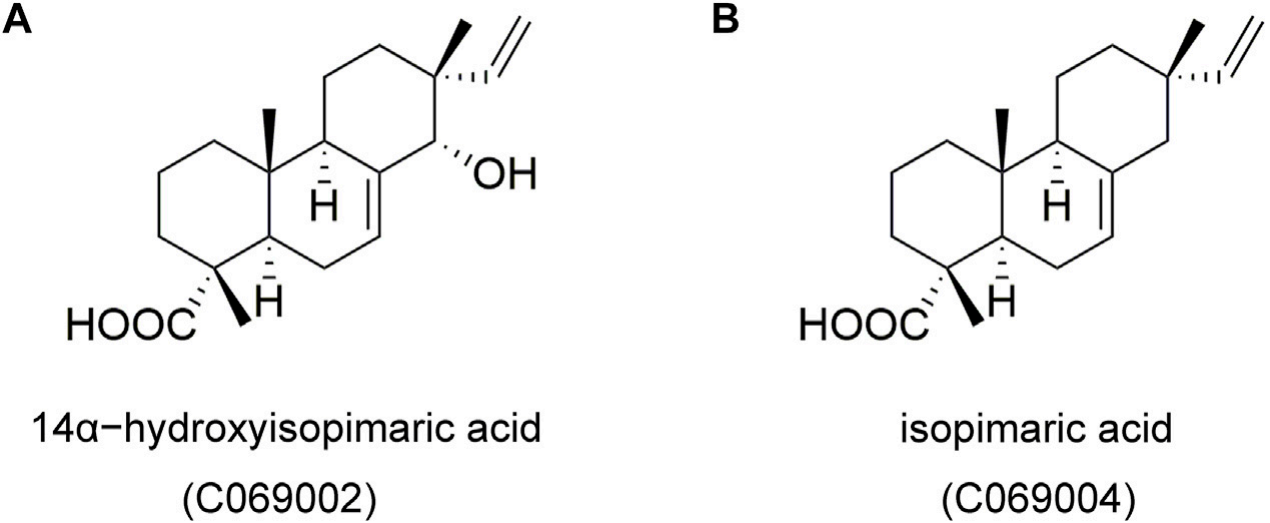
Chemical structures of two isolated compounds, 14α-hydroxyisopimaric acid (C069002) **(A)** and isopimaric acid (C069004) **(B)**, from the flowers of the *Callicarpa rubella* Lindl.

**FIGURE 3:**
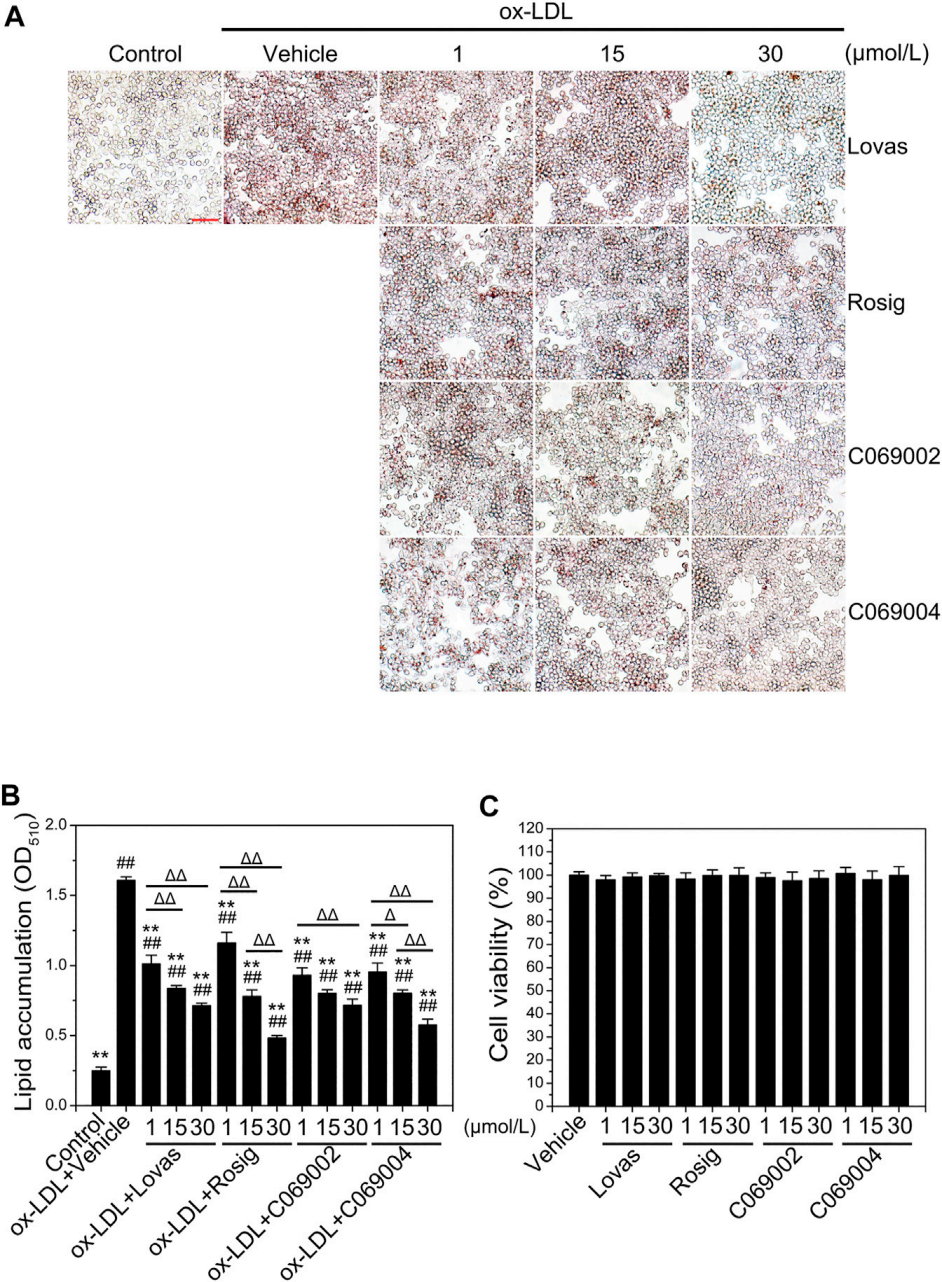
Diterpenoids alleviate ox-LDL-induced intracellular lipid accumulation and have no effect on macrophage cell viability. **(A)** RAW264.7 cells were treated with ox-LDL (80 μg/mL) and different concentrations of lovastatin, rosiglitazone or diterpenoids (C069002 and C069004) for 24 h. Lipid accumulation was determined by oil red O (ORO) staining (×400 magnification). Scale bar = 50 μm. **(B)** Quantitative results were obtained by measuring the eluted ORO **(A)** using a spectrophotometer. **(C)** Diterpenoids have no effect on macrophage cell viability. RAW264.7 cells were incubated with different concentrations of lovastatin, rosiglitazone or diterpenoids (C069002 and C069004) respectively for 24 h and then incubated with MTT for a further 4 h. Cell viability was measured by the absorbance of dissolved MTT crystals using an ELISA reader. Data are shown as mean ± SD from three independent experiments. Significance is presented as *p < .05, **p < .01 *versus* the vehicle (DMSO) group; ^#^ *p* < .05, ^##^ *p* < .01 *versus* the control group, and ^Δ^ *p* < .05, ^ΔΔ^ *p*<.01 between the indicated groups. Lovas: lovastatin; Rosig: rosiglitazone.

Subsequently, the potential effects of diterpenoids on macrophage foam cell formation were detected by treating RAW264.7 cells with different concentrations of two compounds [C069002 (purity: 98.66%) and C069004 (purity: 98.64%)] isolated from the flowers of *Callicarpa rubella* Lindl. (Figures 2A, B and Supplementary Figure S1, S2) for 24 h. Oil red O staining showed that diterpenoids (C069002 and C069004) treatment significantly reduced ox-LDL-induced intracellular lipid accumulation in RAW264.7 macrophages, similar to lovastatin and rosiglitazone (Figures 3A, B). Correspondingly, we found that lovastatin, rosiglitazone, and the diterpenoids (C069002 and C069004) could also prominently reduce the intracellular TC and TG contents (Supplementary Figure S3A, B). In addition, intracellular lipid droplet content was remarkably diminished over time after diterpenoid treatment (Supplementary Figure S4A, B). All the above results indicated that diterpenoids could significantly inhibit ox-LDL-induced macrophage foam cell formation. In the MTT assay, there were no apparent cytotoxic effects in RAW264.7 macrophages treated with C069002 or C069004 at the indicated concentrations (Figure 3C).

### 3.2 Diterpenoids promote cholesterol efflux from macrophage

**FIGURE 4:**
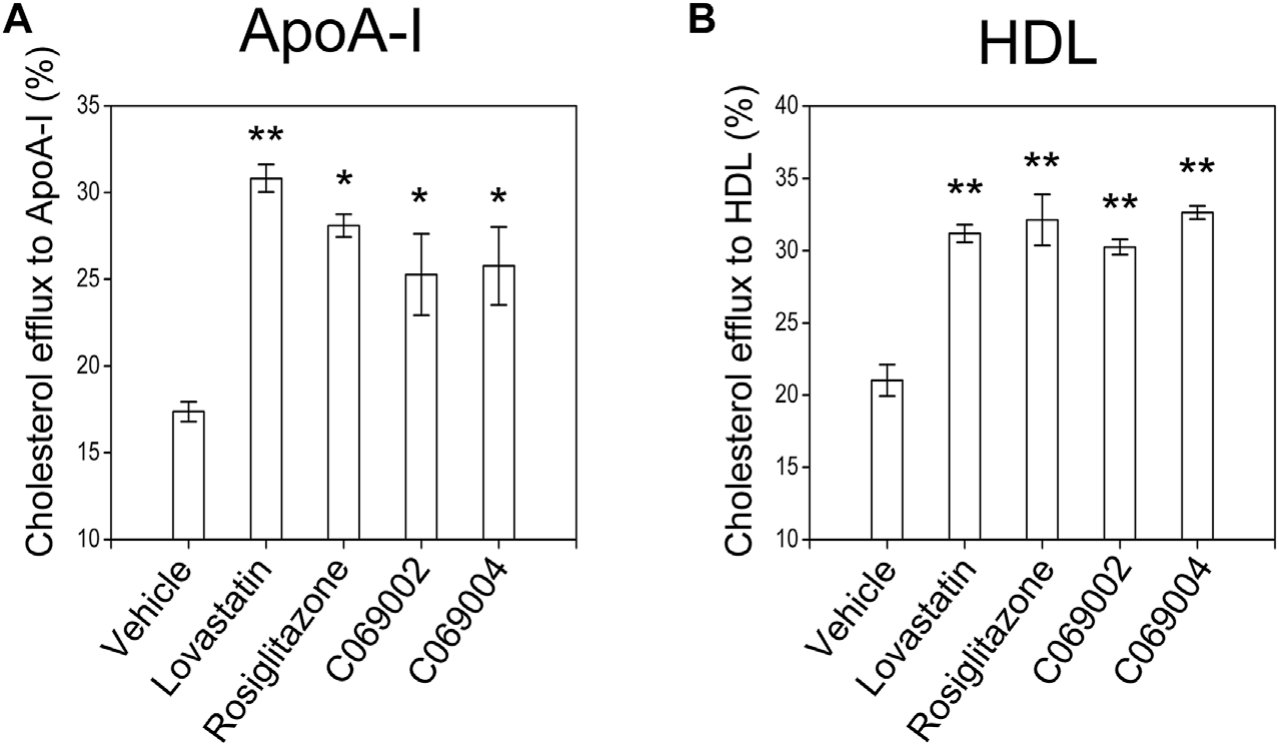
Diterpenoids promote cholesterol efflux in RAW264.7 macrophages. RAW264.7 macrophages were preincubated with 22-NBD-cholesterol for 24 h, after which the cells were washed with PBS and incubated with the indicated drugs and chemical compounds (15 μmol/L). After 18 h, **(A)** 10 μg/mL ApoA-I or **(B)** 50 μg/mL HDL was added, and the incubation continued for 6 h at 37°C. The amounts of cholesterol in the medium and cells were measured. The relative 22-NBD-cholesterol efflux to ApoA-I or HDL induced by the diterpenoids was calculated. Similar results were obtained in three independent experiments. Data are shown as the mean ± SEM from one of the three independent experiments. Significance is presented as *p < .05 and **p < .01 *versus* the vehicle (DMSO) control.

Next, the cholesterol efflux capacity of diterpenoid-treated RAW264.7 macrophages was evaluated. ApoA-I (10 μg/mL) or HDL (50 μg/mL) was added to the medium to promote cholesterol efflux. Similar to the positive controls (lovastatin and rosiglitazone), both C069002 and C069004 significantly increased cholesterol efflux to ApoA-I or HDL and reduced the cellular cholesterol concentration in this cell line (Figures 4A, B).

### 3.3 Diterpenoids induce ABCA1 and ABCG1 expression *in vitro*

**FIGURE 5:**
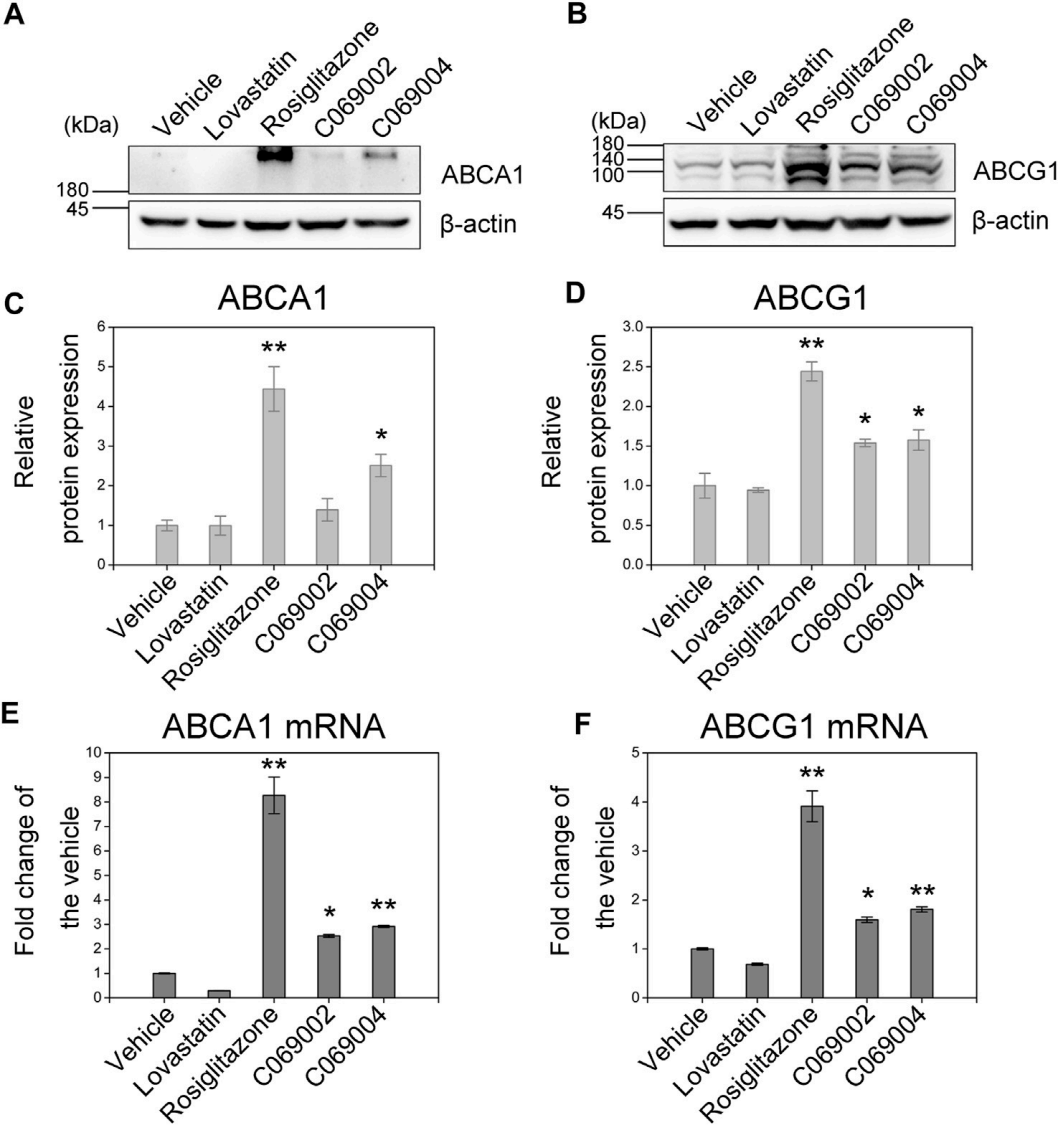
Diterpenoids increase ABCA1 and ABCG1 expression. RAW264.7 macrophages were treated with 15 μmol/L indicated drugs and chemical compounds for 24 h. Cell lysates were collected, and the protein levels of PPARγ **(A)** and LXRα **(B)** were determined by western blotting. Corresponding quantitative results are shown as the mean ± SEM from three independent experiments **(C,D)**. Cellular RNA was collected for quantitative real-time PCR (qRT‒PCR) analysis. Data are shown as the mean ± SEM from three independent experiments **(E,F)**. Significance is presented as *p < .05 and **p < .01 *versus* the vehicle (DMSO) control.

To validate whether the increase in cholesterol efflux to ApoA-I or HDL is mediated by ABCA1 and ABCG1, respectively, the expression of ABCA1 and ABCG1 in murine macrophages treated with diterpenoids were detected by western blotting and quantitative RT-PCR. The results showed that the diterpenoid C069004 significantly increased the protein expression levels of ABCA1 and ABCG1 in RAW264.7 macrophages. The diterpenoid C069002 could also enhance the protein expression level of ABCG1 but had a slight effect on the protein expression level of ABCA1 (Figures 5A–D). However, both compounds prominently increased the ABCA1 and ABCG1 mRNA levels (Figures 5E, F). Thus, these data indicated that diterpenoids could enhance the expression of ABCA1 and ABCG1 in RAW264.7 macrophages. Interestingly, a greater effect on the upregulation of ABCA1 and ABCG1 expression was observed when the cells were stimulated with rosiglitazone, whereas another positive control, lovastatin, hardly affected the protein and mRNA levels of ABCA1 and ABCG1 in RAW264.7 macrophages (Figure 5).

### 3.4 Diterpenoids upregulate LXRα and PPARγ expression in macrophages

**FIGURE 6:**
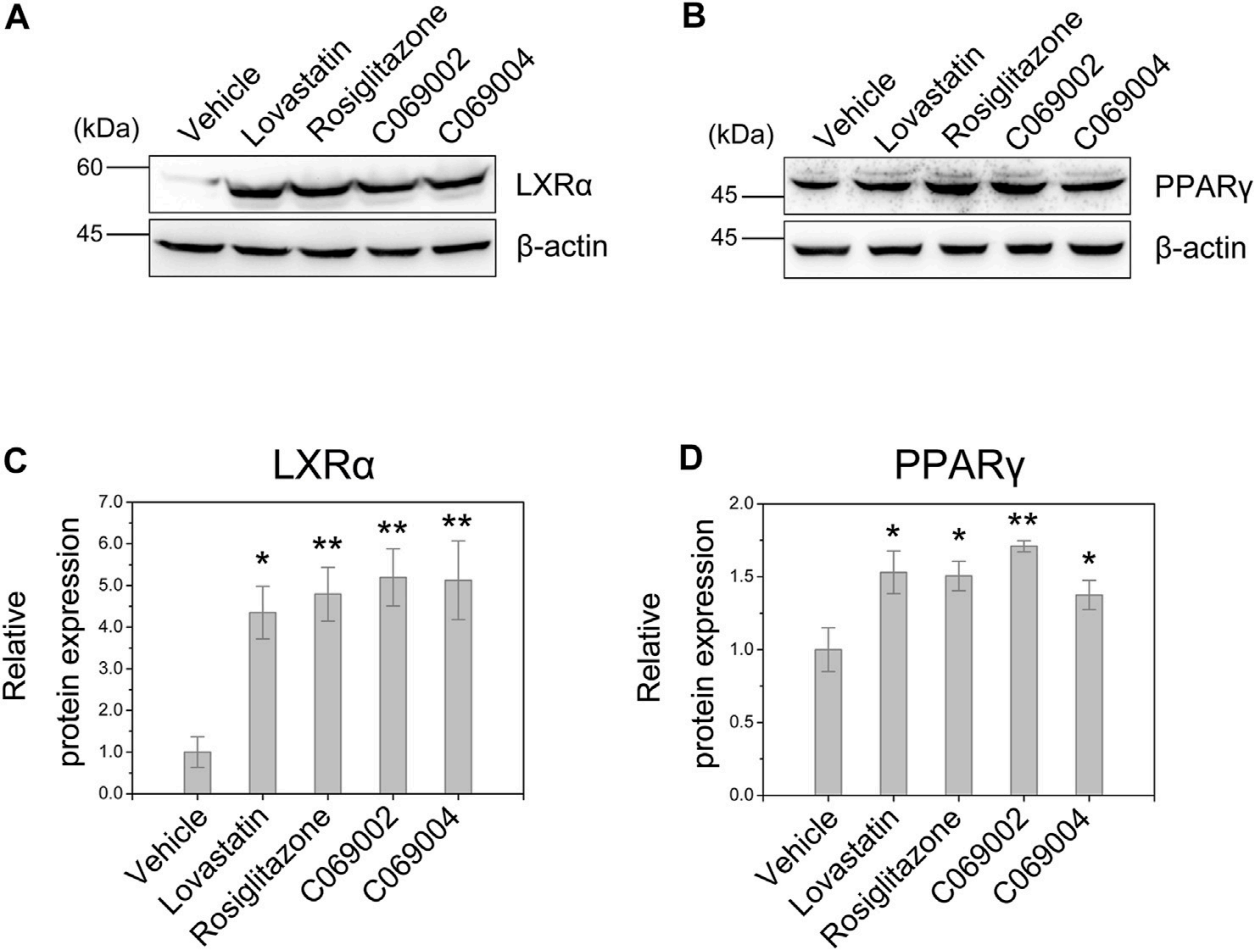
Diterpenoids enhance PPARγ and LXRα expression. RAW264.7 macrophages were treated with 15 μmol/L indicated drugs and chemical compounds for 24 h. Cell lysates were collected, and the protein levels of PPARγ **(A)** and LXRα **(B)** were determined by western blotting. Corresponding quantitative results are shown as the mean ± SEM from three independent experiments **(C,D)**. Significance is presented as *p < .05 and **p < .01 *versus* the vehicle (DMSO) control.

In RAW264.7 macrophages, C069002, C069004, lovastatin, and rosiglitazone treatment significantly upregulated the protein expression of LXRα (Figures 6A, B). Additionally, considering the regulatory role of PPARγ on the LXRα signaling, we further investigated the expression of PPARγ in RAW264.7 macrophages and found that treatment with either diterpenoids (C069002 or C069004) or positive control drugs (lovastatin or rosiglitazone) could increase the protein expression of PPARγ (Figures 6C, D), which suggesting that diterpenoids positively regulate PPARγ/LXRα signaling to enhance ABCA1 and ABCG1 expression.

### 3.5 Diterpenoids activate the PPARγ/LXRα/ABCA1 signaling pathway in macrophage-derived foam cells

**FIGURE 7:**
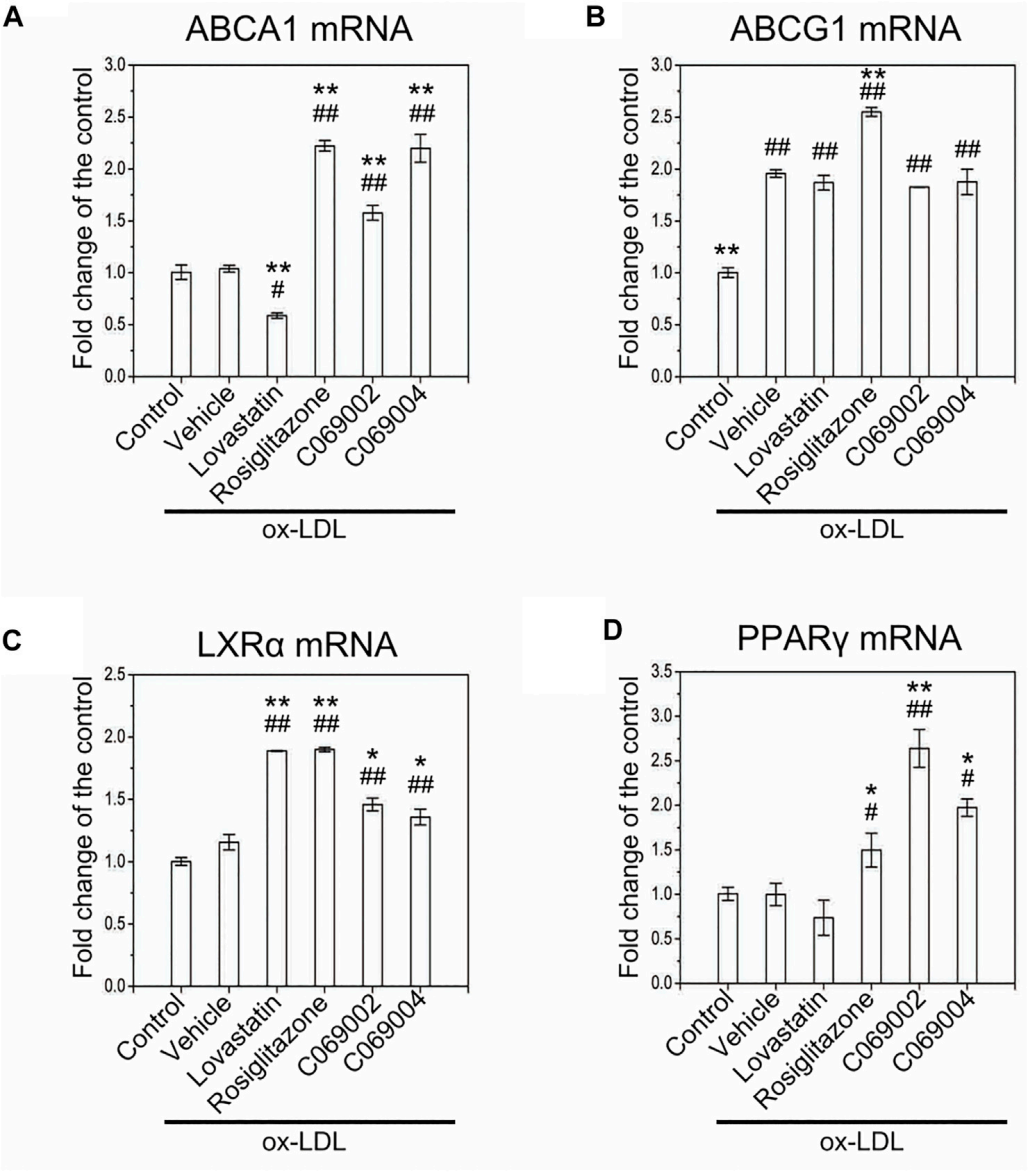
Treatment with diterpenoids increases the mRNA expression of ABCA1, LXRα, and PPARγ in ox-LDL-induced macrophage foam cells. RAW264.7 macrophages were treated with 15 μmol/L indicated compounds and 80 μg/mL ox-LDL for 24 h. Total RNA was extracted from the cells for quantitative real-time PCR (qRT‒PCR) analysis of the mRNA levels of ABCA1 **(A)**, ABCG1 **(B)**, LXRα **(C)** and PPARγ **(D)**. Data are shown as the mean ± SEM from three independent experiments. Significance is presented as *p < .05, **p < .01 *versus* the vehicle (DMSO) group and ^#^ *p* < .05, ^##^ *p* < .01 *versus* the control group.

To further identify the detailed mechanism by which diterpenoids modulate intracellular cholesterol efflux and attenuate ox-LDL-induced macrophage foam cell formation, we examined the expression of ABCA1, ABCG1, LXRα, and PPARγ in RAW264.7 macrophages treated with diterpenoids or positive control drugs while simultaneously being exposed to ox-LDL for 24 h. In response to ox-LDL stimulation, the expression of ABCG1 was induced in RAW264.7 macrophages, and the mRNA level of LXRα was slightly increased concurrently. Moreover, compared to the vehicle control, treatment with diterpenoids or rosiglitazone significantly elevated the mRNA levels of ABCA1, LXRα, and PPARγ in macrophage-derived foam cells induced by ox-LDL (Figures 7A, C, D). However, treatment with diterpenoids did not enhance ox-LDL-induced expression of ABCG1 at the mRNA level (Figure 7B). These results suggested that diterpenoids protect against ox-LDL-induced macrophage foam cell formation probably through ABCA1-mediated cholesterol outflow. Intriguingly, although lovastatin could also upregulate the mRNA expression of LXRα (Figure 7C), it had little effect on the expression of ABCG1 or PPARγ (Figures 7B, D) and even to some extent repressed the expression of ABCA1 in the presence of ox-LDL (Figure 7A), suggesting that lovastatin may utilize some other particular mechanism to perform its function in promoting cholesterol efflux from macrophages.

## 4 Discussion

In the current study, we identified two natural diterpenoid molecules, namely, 14*α*-hydroxyisopimaric acid (C069002) and isopimaric acid (C069004), isolated from the flowers of *Callicarpa rubella* Lindl., with remarkable inhibitory effects on macrophage foam cell formation. Further studies showed that these two molecules could facilitate cholesterol efflux from RAW264.7 macrophages to ApoA-I and HDL. Since ABCA1 and ABCG1 are major transporters involved in cholesterol efflux from macrophages and play a vital role in maintaining cellular cholesterol homeostasis (Li et al., 2016), we next inspected the expression of ABCA1 and ABCG1 in RAW264.7 macrophages upon diterpenoid (C069002 or C069004) treatment and found that diterpenoids significantly induced the expression of ABCA1 and ABCG1 proteins and mRNAs. LXRα signaling induces the expression of many genes involved in cholesterol transport, including *ABCA1* and *ABCG1* (Li et al., 2016; Chen et al., 2020). In addition, the LXRα gene itself is a direct target of PPARγ, and these two nuclear receptors cooperate in the regulation of macrophage ABCA1 expression and the control of cholesterol efflux (Chawla et al., 2001; Du et al., 2021). Here, we demonstrated that in RAW264.7 macrophages, diterpenoids highly upregulated LXRα and PPARγ gene expression. Moreover, the diterpenoids still increased the mRNA transcription levels of the *ABCA1*, *LXRα*, and *PPARγ* genes in the presence of ox-LDL, further illustrating that the regulatory mechanism of diterpenoids on cholesterol efflux was probably through activating the PPARγ-LXRα-ABCA1 cascade.

In this study, to ascertain the underlying mechanism by which the diterpenoids (C069002 or C069004) attenuated macrophage foam cell formation, we used two clinically applied antiatherosclerotic drugs, lovastatin and rosiglitazone, as positive controls. Lovastatin, an inhibitor of 3-hydroxy-3-methylglutaryl coenzyme A (HMG-CoA) reductase, is known to be a potent cardiovascular drug that lowers cholesterol (Eschenhagen and Laufs, 2021). In addition, increasing evidence has highlighted the antiatherosclerotic effect of lovastatin, owing to its pleiotropic inhibitory effects on inflammation, oxidative stress, endothelial dysfunction, thrombosis, macrophage growth, and smooth muscle cell migration and proliferation (Mihos et al., 2014; Jiang et al., 2019). Interestingly, our data showed that lovastatin treatment obviously inhibited RAW264.7 macrophage formation and significantly increased cholesterol efflux to ApoA-I or HDL; moreover, it could induce intracellular LXRα expression. However, no apparent increase in the expression of ABCA1 or ABCG1 at both the protein and mRNA levels was found during lovastatin treatment, regardless of the presence or absence of ox-LDL. These results imply that lovastatin possibly adopts other transporters to promote cellular cholesterol outflow. Rosiglitazone is an orally active antidiabetic drug in the thiazolidinedione drug class. It functions by binding to the PPARγ receptor as an agonist, where it inhibits the progression of atherosclerosis in patients (Kelly and Bank, 2007). Additionally, rosiglitazone has been shown to exert its antiatherogenic effects by inhibiting perivascular mast cell activation, attenuating endothelial injury, improving serum HDL quality and function, and promoting macrophage cholesterol efflux (Li et al., 2015; Li et al., 2019; Xu et al., 2019). Regarding the control of cholesterol efflux, rosiglitazone accelerates cholesterol removal from macrophages by activating the PPARγ-LXRα-ABCA1/G1 pathway (Chawla et al., 2001; Ren et al., 2019). This observation is consistent with our finding that rosiglitazone treatment significantly increased the expression of PPARγ, LXRα, and ABCA1/G1 and promoted cholesterol efflux from RAW264.7 macrophages. Similarly, we found that treatment with diterpenoids (C069002 or C069004) could upregulate PPARγ, LXRα, and ABCA1 expression in RAW264.7 macrophages regardless of the presence or absence of ox-LDL. These findings suggest that diterpenoids and rosiglitazone probably share a similar mechanism for promoting cholesterol efflux, but whether these diterpenoids (C069002 and C069004) are direct agonists or ligands for PPARγ or LXRα or their common heterodimer partner RXR remains elusive and requires further investigation in future studies. Given that LXRs, ABCA1, and ABCG1 are key regulators of the RCT pathway (Zhang et al., 2021), it could be expected that the diterpenoids accelerate the rate of RCT from atherosclerotic lesions through activation of LXRα signaling.

Several limitations should be mentioned. First, the *in vivo* cholesterol efflux effect of the diterpenoids (C069002 and C069004) was not assessed in the current study. Second, the influence of the diterpenoids on another transporter, scavenger receptor BI (SR-BI), localized in the macrophage and hepatocyte membranes, which simultaneously participates in the initial (i.e., cholesterol efflux from macrophages) and final (i.e., selective uptake of cholesteryl ester by hepatocytes) RCT steps, was not evaluated. Third, the effects of diterpenoids on other steps of the RCT pathway, including the synthesis of cholesteryl ester molecules and the selective uptake of lipid molecules by hepatocytes, require further evaluation. Despite these limitations, our study clearly shows the promising atheroprotective effects of diterpenoids.

In conclusion, we identified two diterpenoid-derivative molecules, that is, 14*α*-hydroxyisopimaric acid (C069002) and isopimaric acid (C069004), extracted and separated from the flowers of *Callicarpa rubella* Lindl., with the atheroprotective effects of enhancing cholesterol efflux and inhibiting macrophage foam cell formation. The enhancement of cholesterol efflux by the diterpenoids (C069002 and C069004) occurs through activation of the PPARγ-LXRα-ABCA1 cascade. Accordingly, our study suggests that diterpenoid compounds may be promising candidates in the treatment of atherosclerosis.

## Data Availability

The original contributions presented in the study are included in the article/Supplementary Material, further inquiries can be directed to the corresponding authors.
