# Supplementary material for: Diterpenoids inhibit ox-LDL-induced foam cell formation in RAW264.7 cells by promoting ABCA1 mediated cholesterol efflux
Source: Front Pharmacol. 2023 Jan 12;14:1066758. doi: 10.3389/fphar.2023.1066758 (PMC9877220; doi:10.3389/fphar.2023.1066758)
Supplement: Supplementary file 1 [file DataSheet1.PDF]

Supplementary Material

Supplementary Figures

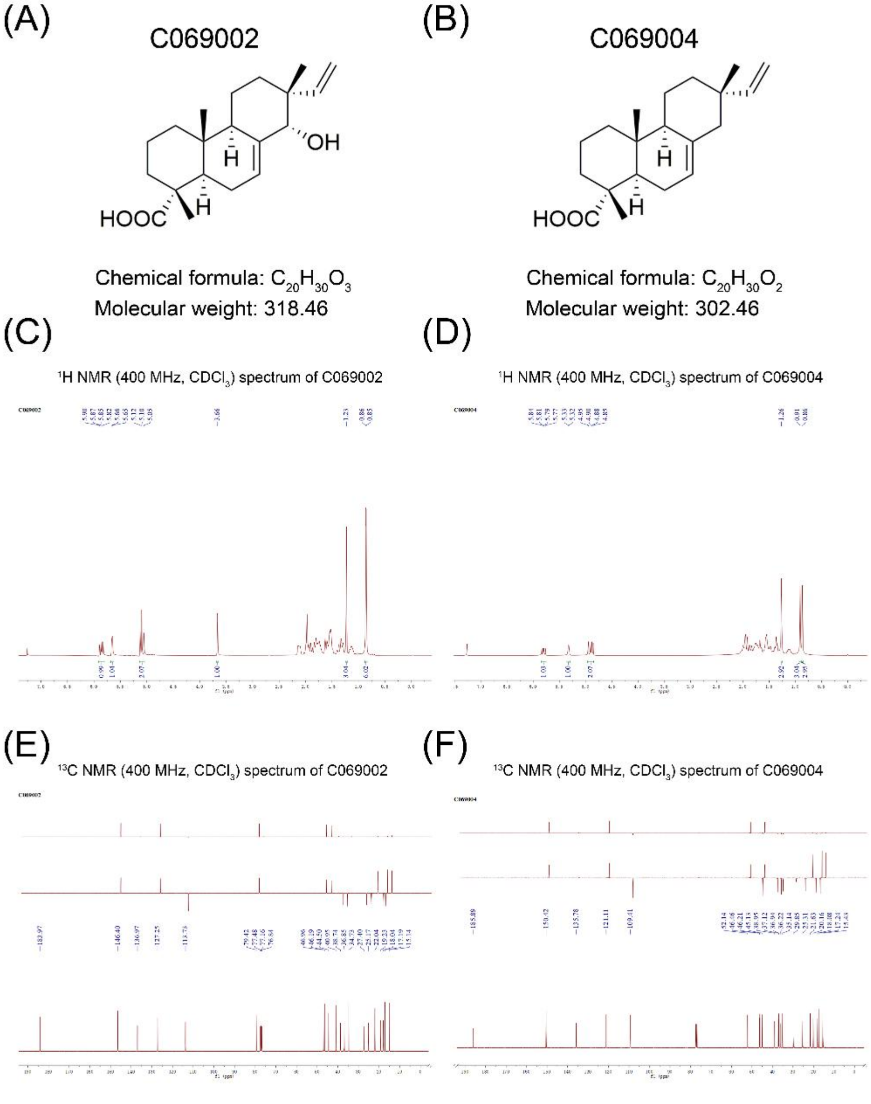

Supplementary Figure 1. Chemical structures of two isolated compounds from the flowers of *Callicarpa rubella*. 14α-Hydroxyisopimaric acid ($C_{20}H_{30}O_3$) (C069002), colorless crystal **(A)**. ^{1}H-NMR (400 MHz, $CDCl_3$): δ 5.86 (dd, 1H, J = 17.6, 10.9 Hz, H-15), 5.66 (d, 1H, J = 4.1 Hz, H-7), 5.09 (t, 2H, J = 14.6 Hz, H-16), 3.66 (s, 1H, H-14), 1.23 (s, 3H, H-19), 0.86 (s, 3H, H-20), 0.85 (s, 3H, H-17) **(C)**. ^{13}C-NMR (100 MHz, $CDCl_3$): δ 184.0 (s, C-18), 146.4 (d, C-15), 137.0 (s, C-8), 127.3 (d, C-7), 113.7 (t, C-16), 79.4 (d, C-14), 47.0 (d, C-9), 46.2 (s, C-4), 44.5 (d, C-5), 40.9 (s, C-13), 38.7 (t, C-1), 36.9 (t, C-3), 34.7 (s, C-10), 27.4 (t, C-12), 25.2 (t, C-6), 22.0 (q, C-17), 19.2 (t, C-11), 18.0 (t, C-2), 17.2 (q, C-19), 15.1 (q, C-20) **(E)**. Isopimaric acid ($C_{20}H_{30}O_2$) (C069004), colorless crystal **(B)**. ^{1}H-NMR (400 MHz, $CDCl_3$): δ 5.80 (dd, 1H, J = 17.5, 10.7 Hz, H-15), 5.31 (d, 1H, J = 3.6 Hz, H-7), 4.90 (dd, 2H, J = 23.6, 14.1 Hz, H-16), 1.26 (s, 3H, H-17), 0.91 (s, 3H, H-20), 0.86 (s, 3H, H-19) **(D)**. ^{13}C-NMR (100 MHz,

$CDCl_3$): δ 185.9 (s, C-18), 150.4 (d, C-15), 135.8 (s, C-8), 121.1 (d, C-7), 109.4 (t, C-6), 52.1 (d, C-9), 46.5 (t, C-14), 46.2 (s, C-4), 45.1 (d, C-5), 38.9 (t, C-1), 37.1 (s, C-13), 36.9 (t, C-3), 36.2 (t, C-12), 35.1 (s, C-10), 25.3 (t, C-6), 21.6 (q, C-17), 20.2 (t, C-11), 18.1 (t, C-2), 17.2 (q, C-19), 15.4 (q, C-20) **(F)**.

(A) C069002, 98.66%

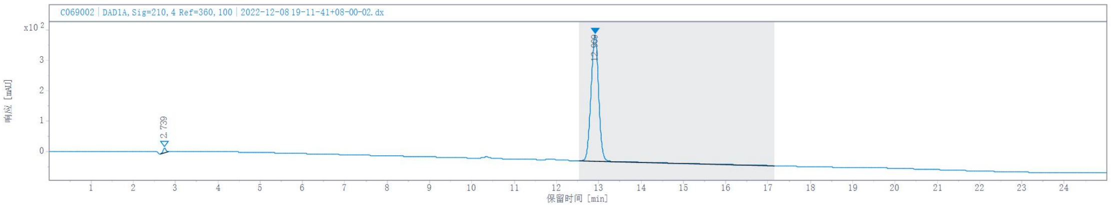

No.	Ret. Time (min)	Peak Name	Area (mAU*min)	Height (mAU)	Area (%)	Type
1	2.736	n.a.	71.17	14.99	1.34	BB
2	12.909	n.a.	5247.67	414.42	98.66	BB
Total			5318.84			

(B) C069004, 98.64%

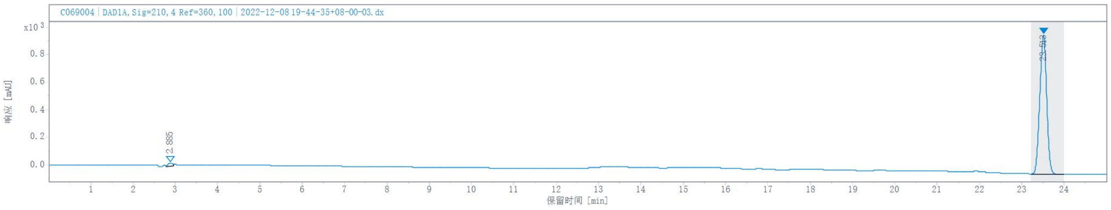

No.	Ret. Time (min)	Peak Name	Area (mAU*min)	Height (mAU)	Area (%)	Type
1	2.885	n.a.	158.52	21.72	1.36	VV
2	23.513	n.a.	11493.36	1010.83	98.641	BB
Total			11651.88			

Supplementary Figure 2. The HPLC analysis results of diterpenoid C069002 and C069004. A high-performance liquid chromatography system (HPLC; Agilent 1260 Infinity II) assembled with a diode array detector was used to quantify the compounds, and the purities detected under 210 nm of **C069002 (A)** and **C069004 (B)** were 98.66% and 98.64%, respectively. An AichromBond-AQ C18 column (5 µm, 4.6 mm* 250 mm) was used as a stationary phase along with the H_2O/CH_3CN (from 40% H_2O/60% CH_3CN to 5% H_2O/95% CH_3CN in 25 mins) as mobile phase for chromatographic separation.

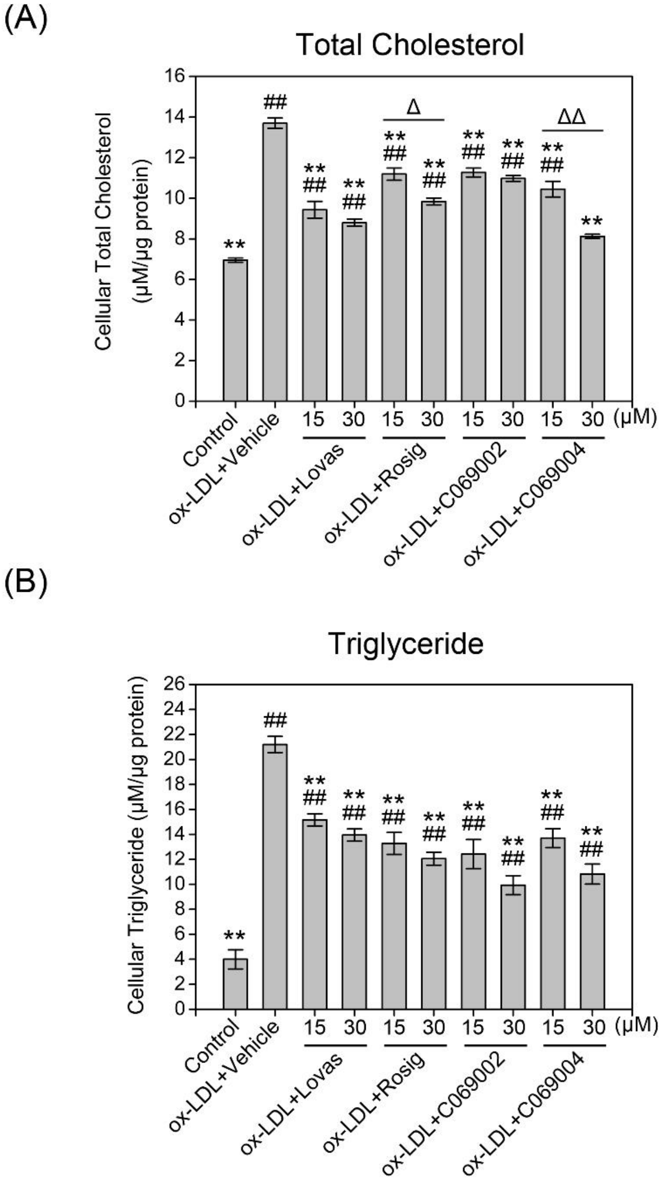

Supplementary Figure 3. Diterpenoids reduce the ox-LDL-induced increases in intracellular TC and TG contents. RAW264.7 cells were treated with 15 or 30 μmol/L indicated drugs and ox-LDL (80 μg/mL) for 24 h. The total cholesterol (TC) **(A)** and triglyceride (TG) contents **(B)** were detected by using their corresponding assay kits. Values represent the mean ± SEM. N=3 in each group. Significance is presented as *p<0.05, **p<0.01 versus the vehicle (DMSO) group; $^{\#}p$<0.05, $^{\#\#}p$<0.01 versus the control group, and $^{\Delta}p$<0.05, $^{\Delta\Delta}p$<0.01 between the indicated groups. Lovas: lovastatin; Rosig: rosiglitazone.

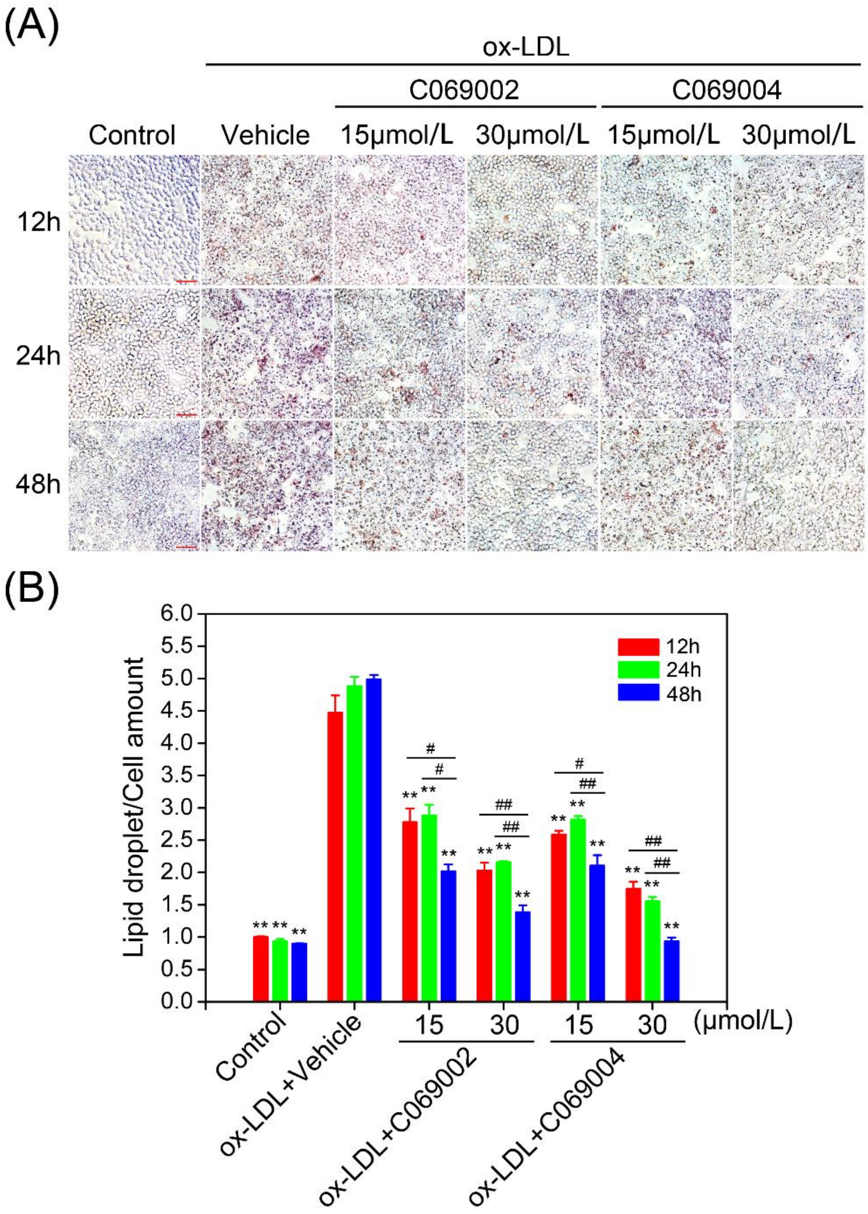

Supplementary Figure 4. Diterpenoids decrease the intracellular lipid droplet content induced by ox-LDL over time in RAW264.7 macrophages. RAW264.7 cells were concurrently treated with different concentrations of diterpenoids (15 or 30 μmol/L) and stimulated with ox-LDL (80 μg/mL) for 12 h, 24 h or 48 h. Intracellular lipids were stained with oil red O and observed by using light microscopy (400× magnification). Scale bar=50 μm **(A)**. The intracellular lipid droplet content was measured by using a spectrophotometer **(B)**. The final lipid droplet content was calibrated based on the cell amount and normalized to the control group at 12 h. Values represent the mean ± SEM. N=3 in each group. Significance is presented as $*p<0.05$, $**p<0.01$ versus the vehicle (DMSO) group, and $^{\#}p<0.05$, $^{\#\#}p<0.01$ between the indicated groups.
